# Bearings in Hip Arthroplasty: Joint Registries vs Precision Medicine: Review Article

**DOI:** 10.1007/s11420-016-9531-7

**Published:** 2016-11-14

**Authors:** Mark J. Pearson, Liam M. Grover, Janet M. Lord, Simon W. Jones, Edward T. Davis

**Affiliations:** 1 Institute of Inflammation and Ageing, University of Birmingham, Birmingham, B15 2TT UK; 2 School of Chemical Engineering, University of Birmingham, Birmingham, B15 2TT UK; 3 The Royal Orthopaedic Hospital, Birmingham, B31 2AP UK

**Keywords:** precision medicine, osteoarthritis, joint registry, total hip replacement

## Abstract

**Background:**

Precision medicine has been adopted in a range of clinical settings where omics data have led to greater characterisation of disease and stratification of patients into subcategories of phenotypes and pathologies. However, in orthopaedics, precision medicine lags behind other disciplines such as cancer. Joint registries have now amassed a huge body of data pertaining to implant performance which can be broken down into performance statistics for different material types in different cohorts of patients. The National Joint Registry of England, Wales and Northern Ireland (NJR) is now one of the largest datasets available. Other registries such as those from Sweden and Australia however contain longer follow-up. Together, these registries can provide a wealth of informative for the orthopaedics community when considering which implant to give to any particular patient.

**Questions/Purposes:**

We aim to explore the benefits of combining multiple large data streams including joint registries, published data on osteoarthritis (OA) pathogenesis and pathology and data concerning performance of each implant material combination in terms of biocompatibility. We believe that this analysis will provide a comprehensive overview of implant performance hopefully aiding surgeons in making more informed choices about which implant should be used in which patient.

**Methods:**

Data from three joint registries were combined with established literature to highlight the heterogeneity of OA disease and the different clinical outcomes following arthroplasty with a range of material types.

**Results:**

This review confirms that joint registries are unable to consider differences in arthritis presentation or underlying drivers of pathology. OA is now recognised to present with varying pathology with differing morbidity in different patient populations. Equally, just as OA is a heterogeneous disease, there are disparate responses to wear debris from different material combinations used in joint replacement surgery. This has been highlighted by recent high-profile scrutiny of early failure of metal-on-metal total hip replacement (THR) implants.

**Conclusions:**

Bringing together data from joint registries, biomarker analysis, phenotyping of OA patients and knowledge of how different patients respond to implant debris will lead to a truly personalised approach to treating OA patients, ensuring that the correct implant is given to the correct patient at the correct time.

**Electronic supplementary material:**

The online version of this article (doi:10.1007/s11420-016-9531-7) contains supplementary material, which is available to authorized users.

## Introduction

The increasing use of joint registry data to guide surgeons in their choice of implant provides a huge opportunity to improve the care that patients receive. The use of these data to identify implants with higher failure rates, such as large bearing metal-on-metal devices, has the potential to significantly reduce harm to our patients. However, the temptation to extrapolate findings within large datasets in an effort to come up with a single “one size fits all” solution must be used with caution.

Over the last few years, there has been an explosion in the use of what is referred to as precision (or personalised) medicine in other areas of health care. This model of patient care moves away from the one size fits all model of health care delivery and provides personalised or precision treatment based on the individual. For example, cancer treatment has been revolutionised by the use of biomarkers to stratify patients into responders and non-responders for specific pharmacological agents. This has been particularly highlighted by AstraZeneca’s development and study of Iressa (Gefitinib) for patients with non-small cell lung cancer. This drug targets the epidermal growth factor receptor (EGFR) and when administered to a large mixed cohort of patients was shown to have poor efficacy. However, 10% of the patient cohort had a mutation in their EGFR, and these patients responded well to treatment [8]. This precision approach therefore improves patient care and reduces health care costs, since those patients who it is predicted will receive either no benefit or at worst a detrimental effect are not administered a costly treatment regimen, in essence providing the right treatment to the right patient.

Within this review, we explore the use of joint registry data in the decision making process with regards to its usefulness and its limitations in selecting the correct bearing for our patients. We also review the evidence that suggests that osteoarthritis (OA) patients are a heterogeneous group and consider the potential for biomarker analysis to provide precision medicine which then assists the clinician in making a more informed choice in selecting the right implant for the right patient.

## Methods

Data from three large joint registries, Swedish Hip Arthroplasty Register, National Joint Registry of England, Wales and Northern Ireland and the Australian Orthopaedic Association National Joint Replacement Registry, regarding implant performance were collated. PubMed literature searches were used to identify articles pertaining to different presentations of OA with regards to environmental and demographic factors which affect OA pathology, thus demonstrating the heterogeneity of the disease. In order to link factors governing OA pathology with implant failure, literature which explore the causal factors which contribute to implant failure were also highlighted.

### International Joint Registries

**Fig. 1:**
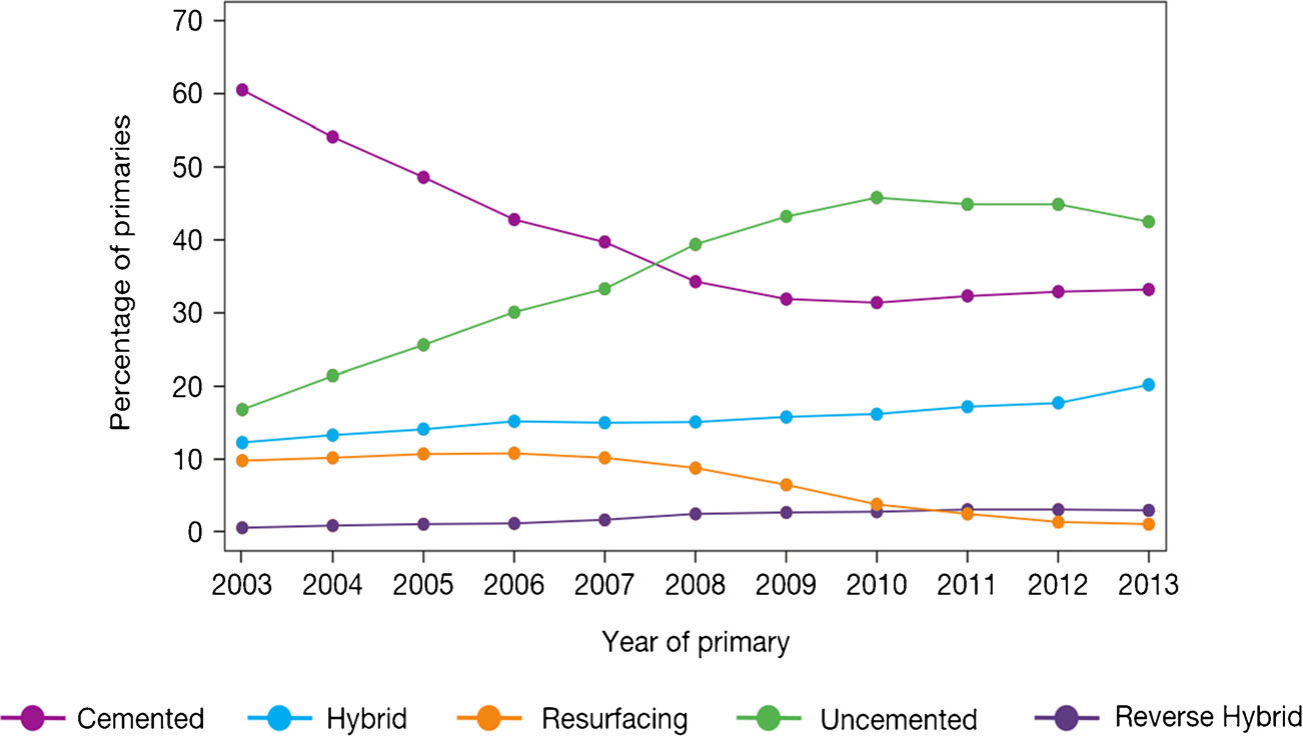
Temporal changes in percentages of each fixation method used in primary hip replacements (from the 2014 report from the National Joint Registry for England, Wales and Northern Ireland, Figure 3.1 www.njrcentre.org.uk).

**Fig. 2:**
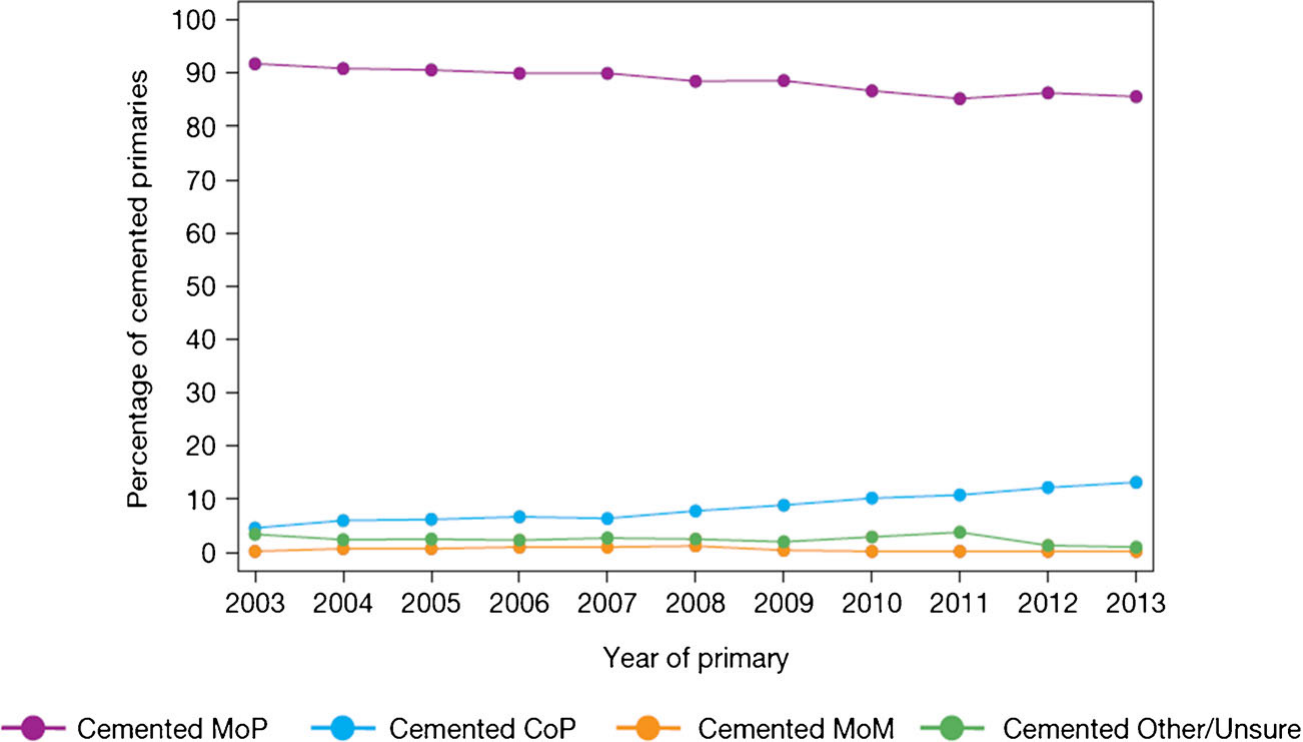
Percentage changes of each bearing surface used in primary hip replacements (from the 2014 report from the National Joint Registry for England, Wales and Northern Ireland, Figure 3.2a www.njrcentre.org.uk).

**Fig. 3:**
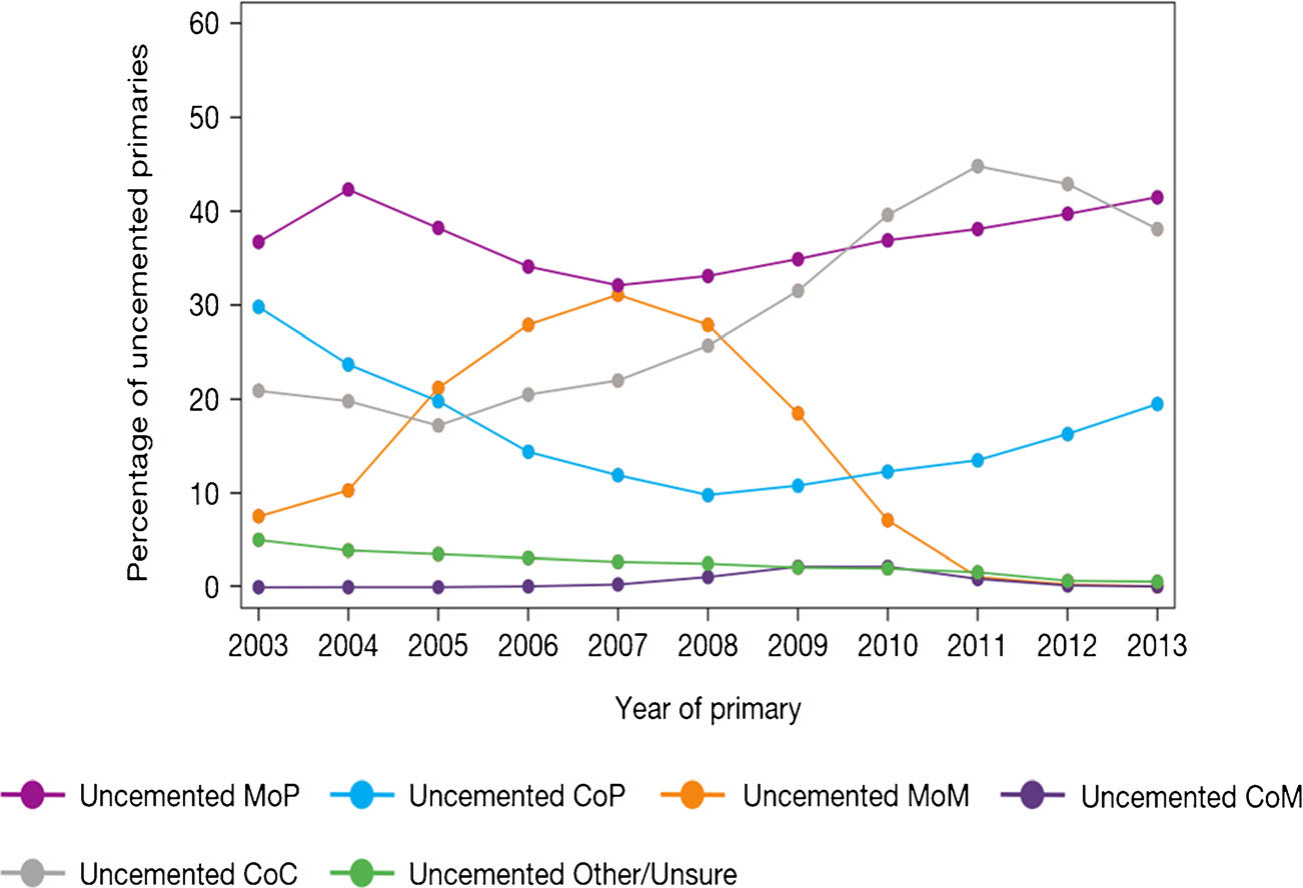
Percentage change in bearing surface used in uncemented total hip replacement (from the 2014 report from the National Joint Registry for England, Wales and Northern Ireland, Figure 3.2b www.njrcentre.org.uk).

**Fig. 4:**
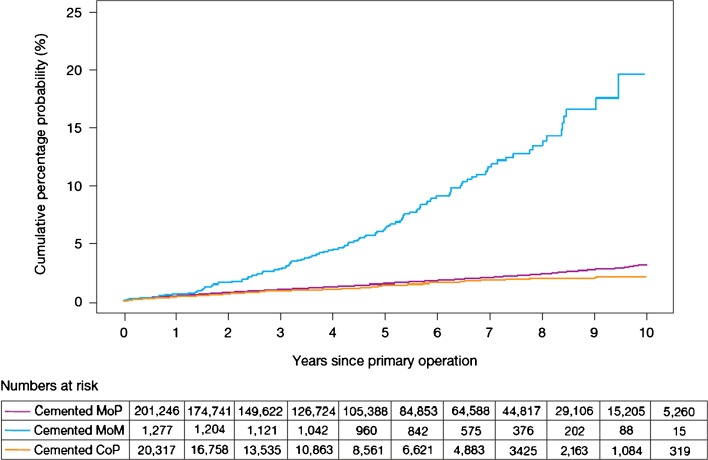
Cumulative probability of revision (Kaplan-Meier estimates) for cemented hips with different bearing surfaces (from the 2014 report from the National Joint Registry for England, Wales and Northern Ireland, Figure 3.4 www.njrcentre.org.uk).

**Fig. 5:**
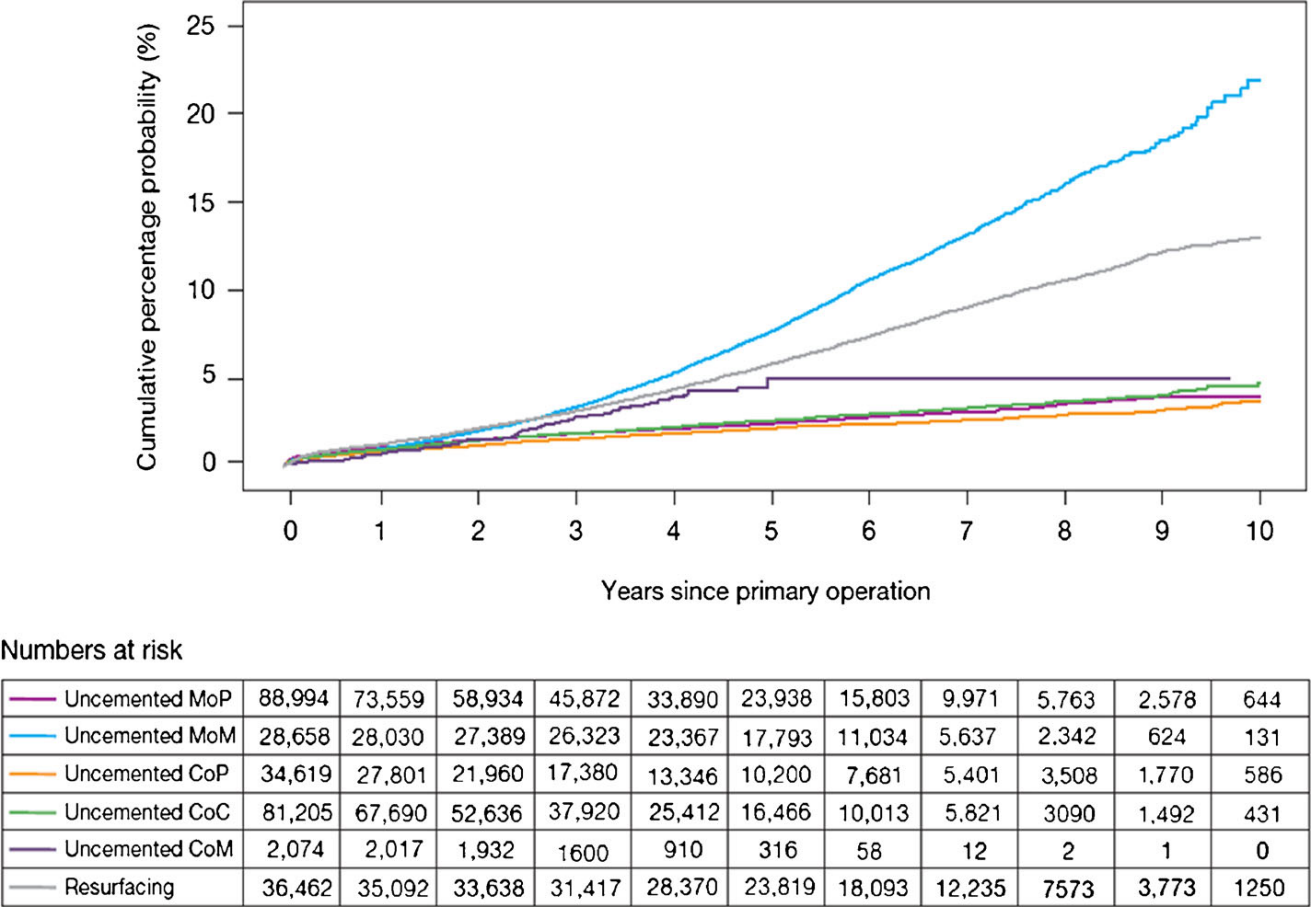
Cumulative probability of revision (Kaplan-Meier estimates) for uncemented hips with different bearing surfaces (from the 2014 report from the National Joint Registry for England, Wales and Northern Ireland, Figure 3.5 www.njrcentre.org.uk).

When considering joint registry data, it is difficult to ignore the contribution from the Swedish Hip Arthroplasty Register [20]. However, the use of different bearing surfaces is relatively limited with the large majority of hip arthroplasty utilising the combination of metal femoral heads with a polyethylene acetabulum. Over the last decade, there has been a gradual migration to the use of cross-linked or modified polyethylene. With the most recent report, the revision rates of the cross-linked polyethylene were found be significantly less than that of conventional polyethylene. However, when confounding factors such as age, gender, femoral head size and acetabular design were considered, the difference failed to reach significance. Data for other bearings in this registry are very limited, and therefore, it is difficult to make comparisons between multiple bearing surfaces. The 2014 report from the National Joint Registry for England, Wales and Northern Ireland (NJR) [2] contained outcome data on over 620,400 hip replacements with a maximum follow-up of 10.75 years. The report provides a wealth of data on the outcome and usage of hip replacements. When the registry was started in 2003, cemented hip arthroplasty was the most common fixation modality. However, more recently, the use of uncemented hip replacements has become more common and is now the main method of fixation of total hip replacements (Fig. 1). The use of hybrid fixation had remained relatively static over the years but has increased slightly, most likely due to outcome data published by the NJR supporting the use of hybrid fixation. The use of different bearing combinations in cemented hip replacement is relatively limited with 90% of the bearing articulations composed of a metal femoral head and a polyethylene acetabular component (Fig. 2). The type of bearing surface used in the uncemented hip replacement is much more varied (Fig. 3). When the registry was initiated, the two most common bearing options were that of a metal femoral head on a polyethylene liner and the use of a ceramic head on a polyethylene liner. Between 2003 and present, the usage of the different bearing combinations has quite dramatically changed. The usage of a metal-on-metal combination increased to its height in 2007 and then decreased following this due to the concerns with respect to metal particle debris. The use of a ceramic-on-ceramic bearings was significantly increased in their usage to a peak in 2011. One explanation of this may have been that ceramic-on-ceramic bearings were marketed as an alternative to metal-on-metal bearings following the decline in use of the latter. Ceramic femoral head on a polyethylene liner usage decreased between 2003 and 2008 but have now gradually increased in their usage, possibly driven by the outcome data produced by the NJR. The use of metal on polyethylene has remained relatively constant and in 2013 was still the most common bearing option for uncemented hip arthroplasty. The NJR provides a wealth of information on the cumulative probability of revision for different bearing combinations, fixation type, age and gender. Overall, the cumulative revision rates for the cemented group appear to show that ceramic on polyethylene may have a slight advantage over that of metal on polyethylene (Fig. 4). Within the uncemented group, the Kaplan-Meier estimates for cumulative probability of revision show that the metal-on-polyethylene, ceramic-on-polyethylene and ceramic-on-ceramic combinations appear to be very tightly clustered with the ceramic on polyethylene appearing to show a slight improvement in revision rates (Fig. 5). One of the present limitations of this registry is that the types of polyethylene are presently not stratified by types of treatment used. This is a concern as other registries have shown a significant advantage of the cross-linked or modified polyethylene.

**Fig. 6:**
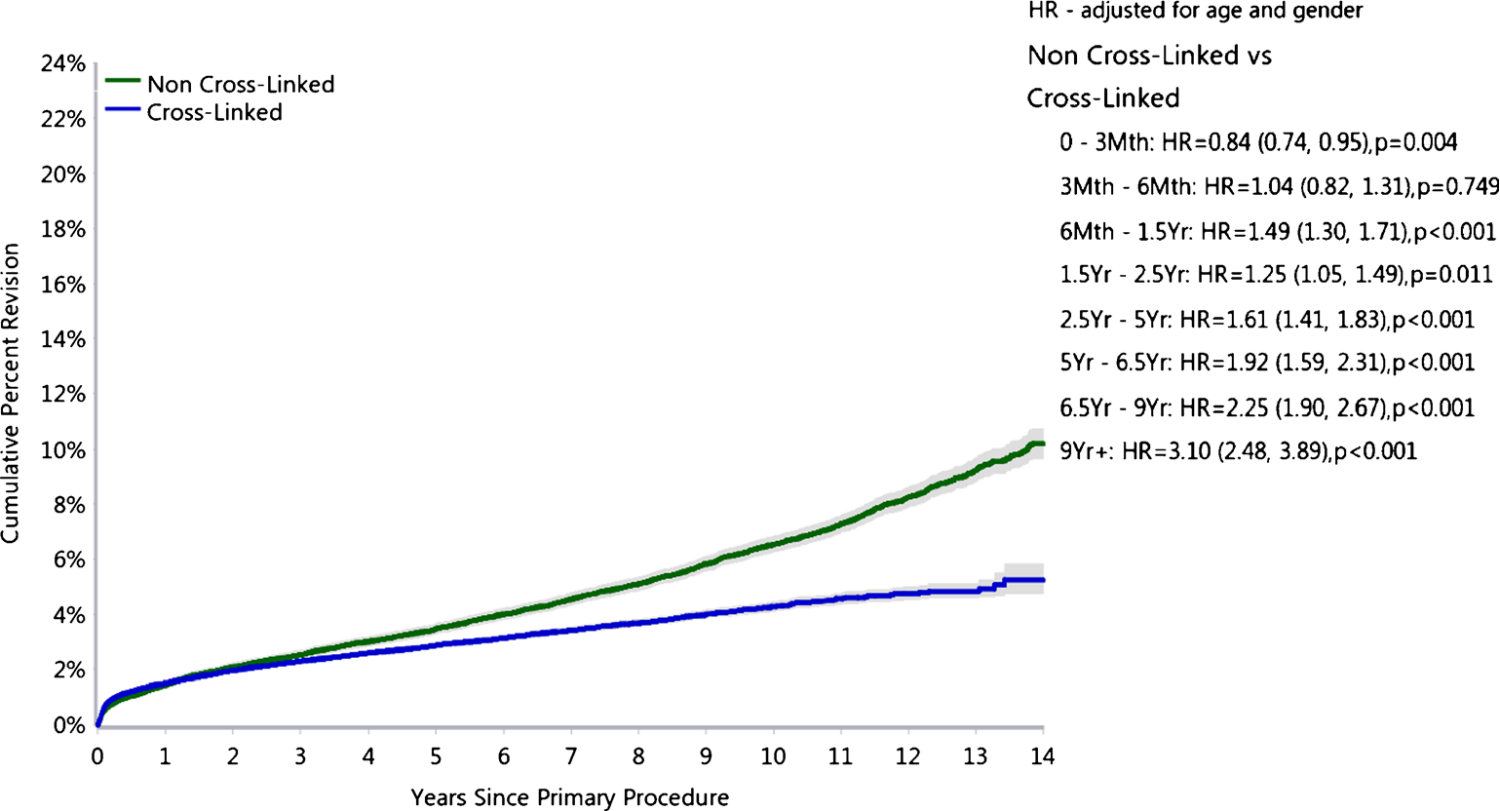
Cumulative percent revision of primary total conventional hip replacement by type of polyethylene (primary diagnosis OA). Figure HT24, Australian Orthopaedic Association National Joint Replacement Registry (AOANJRR). Hip and Knee Arthroplasty Annual Report 2015. Adelaide: Australian Orthopaedic Association (AOA), 2015.

**Fig. 7:**
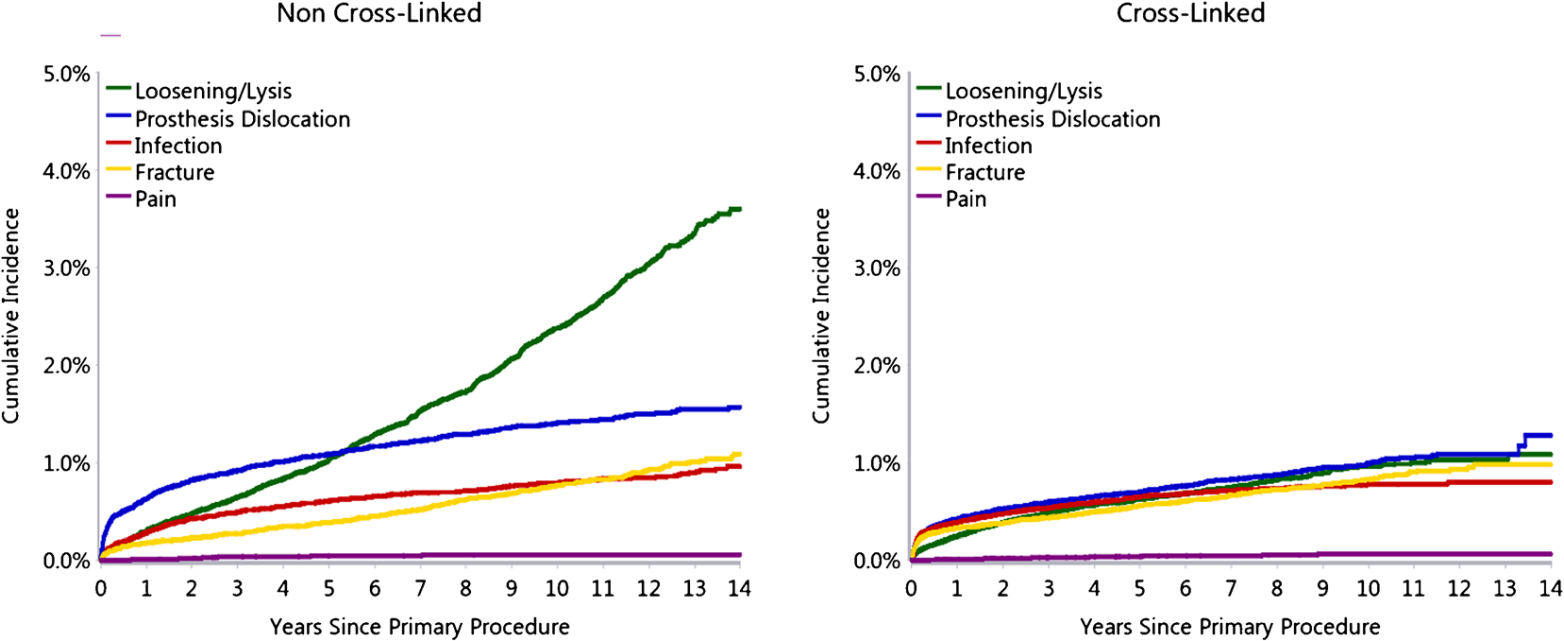
Cumulative incidence revision diagnosis of primary total conventional hip replacement by type of polyethylene (primary diagnosis OA). Figure HT25, Australian Orthopaedic Association National Joint Replacement Registry (AOANJRR). Hip and Knee Arthroplasty Annual Report 2015. Adelaide: Australian Orthopaedic Association (AOA), 2015.

The Australian Orthopaedic Association National Joint Replacement Registry (AOANJRR) [7] was started in 1999, and the annual report published in 2014 contains data on 280,522 primary total hip replacements. The report provides the percentage revision rates of an array of different bearing surfaces. The combination of a ceramicised metal femoral head with a cross-linked polyethylene liner has consistently shown the lowest revision rates (Fig. 6). The use of a metal femoral head or a ceramic femoral head combined with a cross-linked polyethylene liner has also shown low revision rates. However, the combination of a ceramic-on-ceramic bearing surface surprisingly shows a slightly higher revision rate than that of any of the other three combinations. The AOANJRR report clearly shows the significant reduction in revision rates when using cross-linked polyethylene when compared to non-cross-linked polyethylene (Fig. 7). This reduction in revision rate appears to be explained by the reduction in revision for loosening/lysis and a reduction in the number of revisions performed for dislocation. The reduction in dislocation rate would be partially explained by the increased confidence in the use of larger head sizes with the cross-linked polyethylene.

## Discussion

**Fig. 8:**
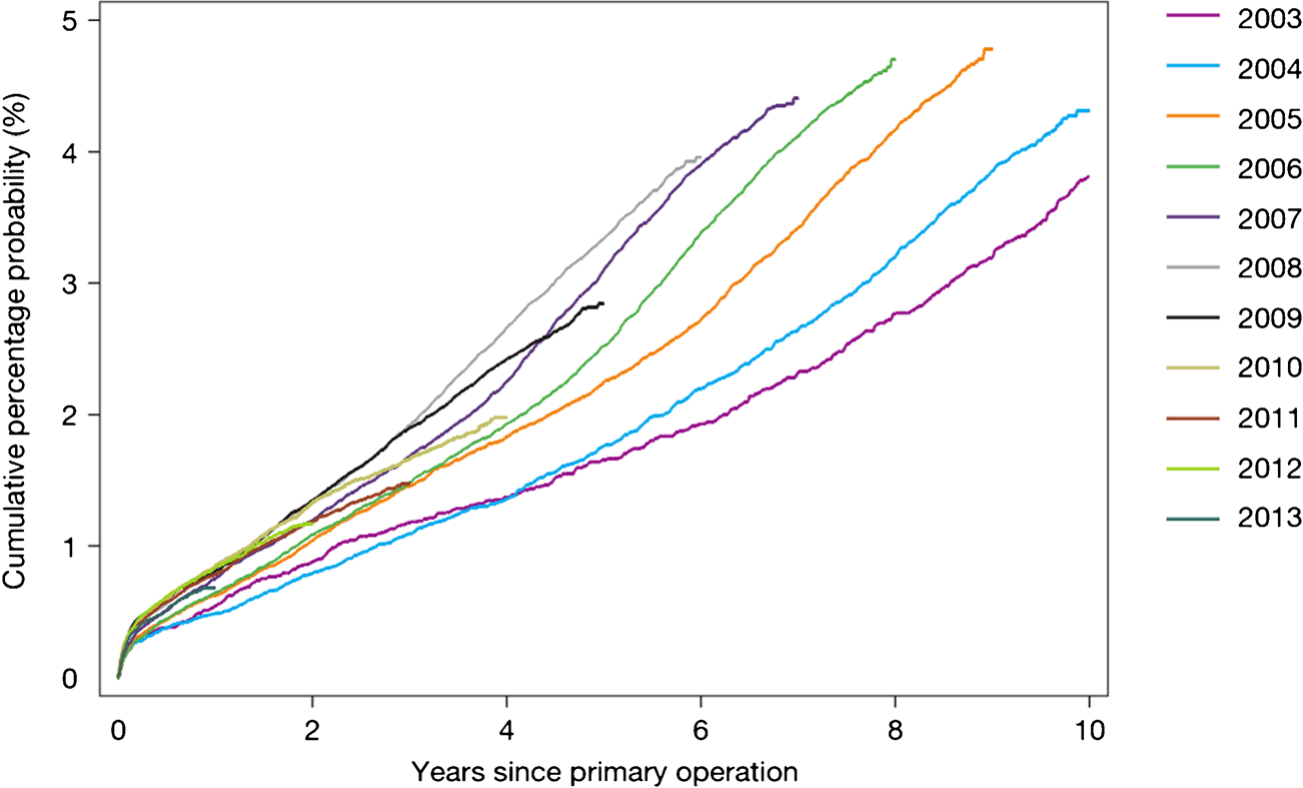
Temporal changes in revision rates: Kaplan-Meier estimates of cumulative percentage probability of revision for each year of primary operation (from the 2014 report from the National Joint Registry for England, Wales and Northern Ireland, Figure 3.3a www.njrcentre.org.uk).

The wealth of information contained within the national joint registries on the outcome of different parameters in hip arthroplasty has the potential to drive significant change in the way that we deliver care. However, the use of these data to inform guidance and policy needs to be treated with caution. This can be shown in the NJR in that the reported revision rates over the years since the commencement of the registry have gradually increased (Fig. 8). One may hypothesise that this is due to the fact that more hips are being revised. However, one alternative hypothesis may be that as the registry has become embedded into clinical practice, the robustness of the reporting of revision operations may have improved. This potential bias would particularly favour those implant combinations that were used at the inception of the registry and detrimental to those introduced more recently. This potential bias would appear to potentially favour cemented fixation with a metal or ceramic femoral head on a polyethylene liner.

When considering how to interpret the outcomes provided by the joint registries, one key aspect that we must not ignore is that of personalised or precision medicine. There is significant pressure when provided with vast datasets to try to provide a one size fits all solution. We must however be cognisant of developments being made in other areas of medicine. Surgeons will often personalise their choice of implant depending on patient characteristics. An example would be that of bone quality and the decision to use a cemented or uncemented femoral component. Good bone quality and a very narrow femoral canal is well suited to a tapered uncemented stem, whereas a porotic femur with a very large femoral canal would be more suited to a cemented femoral component. A step further from this type of personalised care is that of precision medicine which includes the use of biomarkers to stratify patients into different patient groups.

Our understanding of OA and the factors which are involved in pathogenesis has progressed beyond considering this to be a wear and tear disease and has led to a wealth of information pertaining to biomarkers being collected from a multitude of patients. These patients reveal further information regarding the prevalence of each biomarker in different patient cohorts. Therefore, it may be prudent to marry these data to those contained in joint registries to further stratify patients and inform surgeons as to the correct implant for the correct patient at the correct time.

### OA Is a Heterogeneous Disease

With the majority of hip arthroplasty performed for osteoarthritis, it is increasingly recognised that OA is a heterogeneous disease [5, 13]. Multiple tissues within the joint are now implicated in pathogenesis of OA, including not just the cartilage but also the subchondral bone, synovium and adipose tissue, and the involvement of each of these tissues in the pathogenesis may be dependent on the particular patient [23]. For example, the association between increased adiposity and OA risk has been reported to be greater in females [11].

Critically, the heterogeneous nature of OA can also be observed in preoperative X-ray radiographs, where the presence of osteophytes, suggesting the involvement of abnormal subchondral bone remodelling, is present in some patients but not others. Together with variations in the degree of joint space narrowing, patients undergoing joint replacement surgery vary from Kellgren Lawrence (KL) grade 1 to KL grade 4 [12], indicating great diversity in the radiological features of OA across patients [6].

Given that inflammation is now increasingly recognised as a key contributor to OA joint pathology, it is significant that MRI and histopathological studies show that the degree of synovitis (synovial inflammation) varies between OA patients [4]. Notably, our preliminary studies suggest that those OA patients who are obese exhibit a more “inflammatory” phenotype than those patients who are of normal weight. Indeed, inflammation associated with the synovium and tissues adjacent to the synovium is more prevalent in obese patients with OA, compared to normal weight OA patients [24]. This increase in inflammation in obese individuals can be partly attributed to our understanding now that adipose tissue is an endocrine organ, capable of releasing cytokines (termed adipokines), which can mediate proinflammatory and ultimately pathological effects on the joint tissues.

The functional role of particular adipokines in OA pathology is still being investigated, but current literature suggests that the adipokines leptin, resistin and visfatin all contribute to inflammation in the joint [14, 15, 18, 25]. Importantly however, differences in both the functional effects in the joint and/or expression of these adipokines in serum and in joint tissues have been reported to be dependent on BMI and gender [18].

This evidence demonstrates that cellular activation varies between OA patients. This naturally leads OA presentation to differ between patients giving rise to a heterogeneous OA phenotype. As will be discussed below, activation of immune cells is, at least in part, responsible for the failure of some arthroplasties in some patients. Patients whose OA phenotype is particularly inflammatory may, therefore, not be suited to some implant types which are known to invoke an inflammatory response.

### OA Joint Implants: One Size Does not Fit All

One particular area of concern in the arthroplasty domain has been the skill of implantation and the familiarity of implants to the surgeon. There is, therefore, a balance between the personalisation of implant selection to best suit the patient and the number of different implants that the surgeon uses to ensure that they remain sufficiently skilled with each device.

In the same way that OA is a heterogeneous disease with different factors affecting disease presentation and progression, the biological response of individuals to implant materials is also diverse.

The cumulative percentage probability of first revision at 10 years is 2–4% for bearing combination such as metal or ceramic on polyethylene and ceramic on ceramic. The exception to this is metal on metal which has a cumulative risk percentage of 22% at 10 years [21]. This discrepancy in longevity between metal on metal and other implants was highlighted by Smith et al. [21]. Despite metal on metal being predicted to be hard-wearing, there is a 50% greater risk of metal-on-metal (MoM) failure compared to that of metal-on-polyethylene 2 years post-surgery. The reason for this high risk of failure is only now beginning to be understood. However, such has been the fallout from the revelation that MoM implants are negatively impacting on the patients who receive them, a large body of work has been undertaken to understand the reason for patients’ adverse reactions to different material types at the molecular level [3, 16, 19, 22]. This work includes examination of components of the implant such as the taper junction and acetabular cup interface [1, 26].

One particular area of research is to examine the way in which the patient’s immune system responds to the burden of challenge from wear debris. Importantly, it is now accepted that CoCrMo alloy debris elicits an immune response which involves both macrophages and T cells [10, 17], along with an increase in proinflammatory cytokine production [17]. Furthermore, metal wear debris becomes coated in protein from intra-tissue fluids and serum, and it is postulated that this coating, which would include immunomodulatory proteins such as complement, could be used by macrophages to recognise and internalise non-biological materials [9].

Indeed, we have recently performed our own study to investigate the role of the immune system in responding to wear debris from metal-on-metal implants and the resulting inflammatory response. Our findings show that individuals will produce profoundly contrasting systemic biological responses to the presence of wear debris from joint implants and that these transcend the macrophage to implicate other lymphocytes such as T cells [17]. Additionally, individuals will produce different cytokine profiles in response to wear debris, and these profiles could be used to prognostically determine the risk of using a particular material type in any individual patient as well as in the treatment of patients who are showing adverse effects from already receiving an inappropriate implant.

Lessons from metal-on-metal THR implants have demonstrated that the preference of the surgeon alone should not be a driver in the determination of treatment for all patients. Vast quantities of data are deposited in joint registries regarding the performance of different implants. These data, coupled with the determination of immune tolerance towards implant materials and biomarker studies to stratify the heterogenic OA patient population, could pave the way for a truly personalised approach to determining which implant a specific patient should be given.

## Electronic supplementary material

Below is the link to the electronic supplementary material.

ESM 1 (PDF 1224 kb)

ESM 2 (PDF 1224 kb)

ESM 3 (PDF 1224 kb)

ESM 4 (PDF 1224 kb)

ESM 5 (PDF 1225 kb)
